# Proteomic analysis reveals a protective role of specific macrophage subsets in liver repair

**DOI:** 10.1038/s41598-019-39007-6

**Published:** 2019-02-27

**Authors:** Wenting Yang, Xinyuan Zhao, Yuandong Tao, Yan Wu, Fuchu He, Li Tang

**Affiliations:** 1 State Key Laboratory of Proteomics, National Center for Protein Sciences, Beijing, Beijing Proteome Research Center, Beijing Institute of Lifeomics, Beijing, 102206 P. R. China; 2 Department of Biochemistry and Molecular Biology, Anhui Medical University, Hefei, Anhui Province 230032 P. R. China

## Abstract

Macrophages are a heterogeneous population of immune cells that play central roles in a broad range of biological processes, including the resolution of inflammation. Although diverse macrophage subpopulations have been identified, the characterization and functional specialization of certain macrophage subsets in inflamed tissues remain unclear. Here we uncovered a key role of specific macrophage subsets in tissue repair using proteomics, bioinformatics and functional analysis. We isolated two hepatic monocyte-derived macrophage subpopulations: Ly6C^hi^CX_3_CR1^lo^ macrophages and Ly6C^lo^CX_3_CR1^hi^ macrophages during distinct phases of acute liver injury and employed label-free proteomics approach to profile the proteome of these cells. We found that the endocytosis- and apoptotic cell clearance-related proteins were specifically enriched in Ly6C^lo^CX_3_CR1^hi^ macrophages at the resolution phase. Intriguingly, 12/15-lipoxygenase (Alox15), the most strongly up-regulated protein in Ly6C^lo^CX_3_CR1^hi^ macrophages, was identified as a specific marker for these macrophages. In co-culture systems, Ly6C^lo^CX_3_CR1^hi^ macrophages specifically induced hepatocyte proliferation. Furthermore, selective depletion of this population in CD11b-diphtheria toxin receptor mice significantly delayed liver repair. Overall, our studies shed light on the functional specialization of distinct macrophage subsets from different phases in the resolution of inflammation.

## Introduction

Inflammation is an adaptive response that is triggered by infection or damage, with the aim of restoring tissue homeostasis^1–3^. However, inadequate or insufficient resolution of inflammation can result in tissue destruction, chronic inflammation and dysregulation of tissue repair, giving rise to fibrosis and cancer. Thus, it is not unexpected that resolution of inflammation is extremely tightly regulated^4,5^. Significant evidence implicates that macrophages play crucial roles in triggering resolution of inflammation through phagocytosis of cellular debris and releasing cytokines and growth factors that stimulate tissue repair and regeneration^6,7^.

After injury, circulating monocytes are abundantly recruited and then differentiate into macrophages as they migrate into the inflammatory sites^8^. Given that macrophages possess a striking functional and phenotypic plasticity, several studies have shown that there are distinct subsets of macrophages during different stages of inflammation and suggest that they may play unique and different roles^6,9^. Using CD11b-diphtheria toxin receptor (DTR) transgenic mice to selectively deplete macrophages at different stages in carbon tetrachloride-induced liver injury, Duffield *et al*. showed that macrophage depletion when liver fibrosis was advanced resulted in amelioration of fibrosis, whereas macrophage depletion during recovery phase, led to a failure of resolution with impaired matrix degradation^10^. During skeletal muscle repair, recruited monocyte-derived macrophages exhibited pro-inflammatory profiles and then converted to anti-inflammatory macrophages which stimulated myogenesis and fiber growth^11^, and disrupting the phenotypic switch of macrophages impaired healing and regeneration^12^. In a mouse model of spinal-cord injury and repair, distinct macrophage populations were found in the traumatized spinal cord. Ly6C^hi^CX_3_CR1^lo^ macrophages homed to the sites of injured tissue in a CCL2 chemokine-dependent manner, while Ly6C^lo^CX_3_CR1^hi^ macrophages trafficked through a distinct path guided by VCAM-1, VLA-A, and CD73^13^. Although distinct macrophage subpopulations have been identified in various organ systems, the characterization and functional specialization of certain macrophage subsets in discrete microenvironment are not fully understood.

Acetaminophen (N-acetyl-p-aminophenol, APAP) overdose can cause severe liver injury and is the most common cause of death due to acute liver failure in many developed countries^14^. Previous studies have demonstrated that a substantial number of monocyte-derived macrophages were recruited into the inflamed liver^15^. Moreover, the resolution of hepatic damage was delayed in monocyte-deficient *Ccr2*^−/−^ mice compared with WT mice^16^. Despite the essential protective effects of macrophages for liver repair, little is known about which macrophage subsets directly induce hepatocyte regeneration.

Mass Spectrometry (MS)-based proteomics is a powerful approach for in-depth characterization of the protein components of biological systems^17^. The proteome of *in vitro* polarized macrophages have been extensively studied^18,19^. However, relatively little is known about the proteomic characteristics of distinct primary macrophage populations in inflamed tissues. Here, we performed a systematic global proteomic comparison of two hepatic monocyte-derived macrophage subpopulations (Ly6C^hi^CX_3_CR1^lo^ macrophages and Ly6C^lo^CX_3_CR1^hi^ macrophages) from distinct phases of acute liver injury. LC-MS/MS analysis of proteomic profiling revealed that the 72 h Ly6C^lo^CX_3_CR1^hi^ macrophages displayed upregulation of many wound healing- and endocytosis-related proteins relative to the 24 h Ly6C^hi^CX_3_CR1^lo^ macrophages. Notably, the functional contribution of Ly6C^lo^CX_3_CR1^hi^ macrophages to liver repair and regeneration was further confirmed in *in vitro* macrophage-hepatocyte co-culture systems and *in vivo* conditional depletion of Ly6C^lo^CX_3_CR1^hi^ macrophages experiments.

## Results

### Experimental workflow for the differential proteomic study on distinct macrophage subpopulations

**Figure 1:**
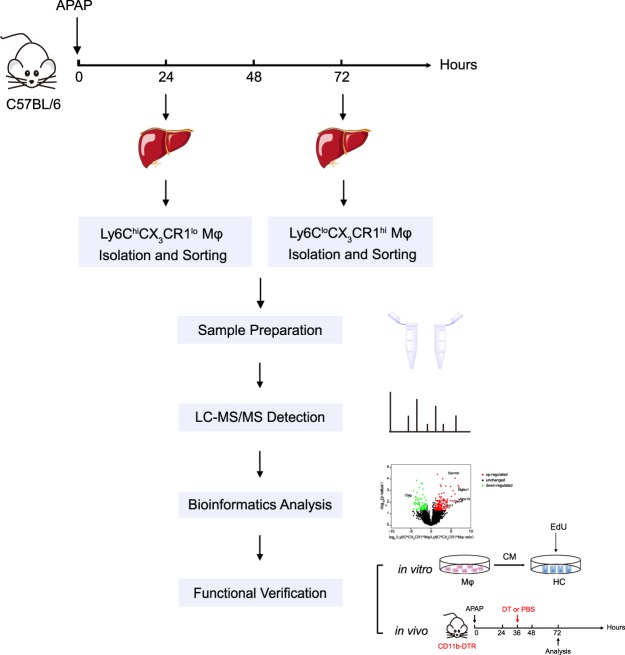
Experimental workflow for the differential proteomic study on distinct macrophage subpopulations.

APAP-induced liver injury displays distinct injury (0–24 h) and resolution (48–72 h) phases and different monocyte-derived macrophage populations have been observed to infiltrate the inflammatory sites^20^. Thus, APAP-induced liver injury provides an instructive model for proteomic analysis of distinct macrophage populations. To explore the functional specialization of distinct hepatic macrophage subsets in APAP-induced liver injury, global label-free quantification (LFQ) proteomics were used. The experimental workflow was shown in Fig. 1. C57BL/6 WT mice were challenged with APAP to induce acute liver injury. Then, primary hepatic leukocytes were isolated and distinct hepatic macrophage populations (Ly6C^hi^CX_3_CR1^lo^ macrophages and Ly6C^lo^CX_3_CR1^hi^ macrophages) were sorted by flow cytometry during the early phase and recovery phase, respectively. Then, the cells were collected and processed for proteomic profiling. Data from proteomics measurements were subjected to comprehensive bioinformatics analysis. Finally, functional validations were performed by both *in vitro* and *in vivo* experiments based on the information and clues obtained from proteomic data.

### Characterization of distinct macrophage subsets in APAP-induced liver injury

**Figure 2:**
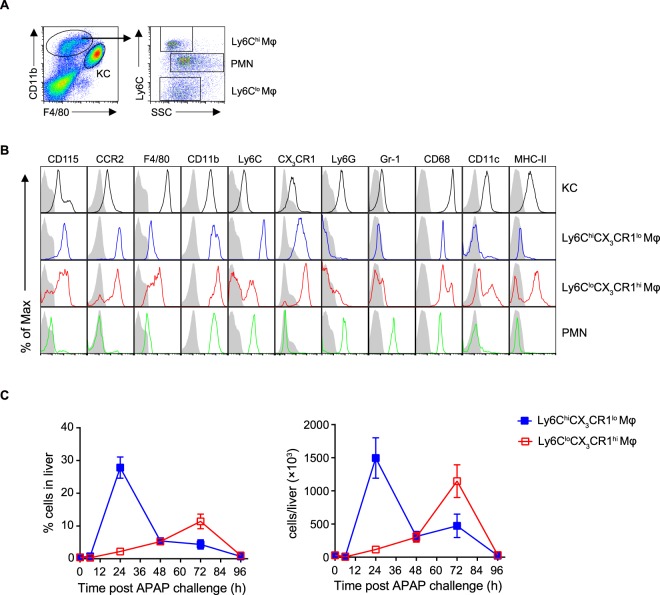
Characterization of distinct macrophage subsets in APAP-induced liver injury. C57BL/6 mice were injected intraperitoneally with APAP at 400 mg/kg (body weight) to induce acute liver injury. (**A**) Flow cytometric analysis of hepatic leukocytes at 48 h after APAP challenge. Liver non-parenchymal cells were identified by first gating for live CD45^+^ leukocytes. Kupffer cells were identified as F4/80^hi^CD11b^lo^. Monocyte-derived macrophages were identified as F4/80^lo^CD11b^hi^. Distinct subsets of monocyte-derived macrophages and neutrophils were identified based on differential Ly6C expression and the FSC/SSC profile. (**B**) Flow cytometric analysis of the indicated populations as gated in (**A**). The shaded histograms represent the unstained controls. (**C**) The percentage and absolute numbers of Ly6C^hi^CX_3_CR1^lo^ and Ly6C^lo^CX_3_CR1^hi^ monocyte-derived macrophages in the livers at each time point were determined by flow cytometry. Data shown are representative of three independent experiments (n = 3/group).

Consistent with previous reports^20,21^, we identified two main monocyte-derived macrophage populations infiltrating in the inflamed liver by flow cytometry: Ly6C^hi^CX_3_CR1^lo^ and Ly6C^lo^CX_3_CR1^hi^ macrophage populations, distinguished by cell surface expression of F4/80, CD11b, Ly6C, CD115, CCR2, CX3CR1, Ly6G, Gr-1, CD68, CD11c and major histocompatibility complex class II (MHC-II) (Fig. 2A,B). Furthermore, dynamic changes in these macrophage subsets throughout the injury and recover phases of inflammation were analyzed. The number of Ly6C^hi^CX_3_CR1^lo^ macrophages increased significantly during the early phase of inflammation, peaked at 24 h and then decreased, whereas Ly6C^lo^CX_3_CR1^hi^ macrophages became the dominant population during the resolution phase (Fig. 2C). Taken together, these results suggest that Ly6C^hi^CX_3_CR1^lo^ macrophages and Ly6C^lo^CX_3_CR1^hi^ macrophages represented the most numerous macrophage population at the early phase and resolution phase of hepatic inflammation, respectively.

### Global proteomics of distinct macrophage subsets from different phases in APAP-induced liver injury

**Figure 3:**
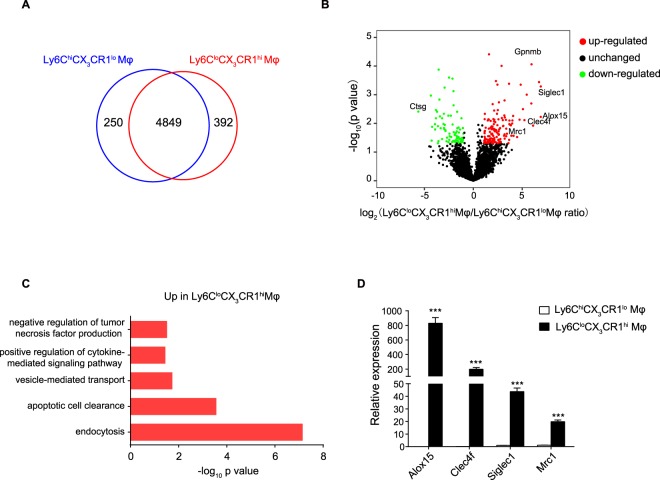
Proteomic profiling of distinct macrophage subsets from different phases in APAP-induced liver injury. (**A**) Macrophage populations during distinct phases from 8–10 mice were used and pooled to acquire proteins for mass spectrometry analysis. Venn diagram of the quantified proteins in 24 h Ly6C^hi^CX_3_CR1^lo^ macrophages and 72 h Ly6C^lo^CX_3_CR1^hi^ macrophages. (**B**) Volcano plot generated by differential analysis of the proteome profiles of 72 h Ly6C^lo^CX_3_CR1^hi^ macrophages compared with those of 24 h Ly6C^hi^CX_3_CR1^lo^ macrophages. Significantly up-regulated proteins were shown as red dots and down-regulated proteins were shown as green dots. The proteins with fold change ≥2 and p < 0.05 were considered to be significant differentially expressed proteins. (**C**) Gene Ontology (GO) enrichment analysis of differentially expressed proteins was classified by their biological functions and arranged according to their statistical significance (-log_10_ p value on x axis) from DAVID. Biological processes involved in immune response were shown. (**D**) Validation of the indicated differentially expressed genes by qPCR, presented relative to *Gapdh*. Data shown are representative of three independent experiments (n = 3/group). Results represent mean ± SEM (*p < 0.05, **p < 0.01, ***p < 0.001).

Having uncovered massive infiltration of distinct monocyte-derived macrophage subsets in injured livers during different phases of inflammation, we performed proteomic profiling of FACS-sorted 24 h Ly6C^hi^CX_3_CR1^lo^ macrophages and 72 h Ly6C^lo^CX_3_CR1^hi^ macrophages using label-free proteomics. The proteins were identified and quantified by Maxquant software. We analyzed four independent biological replicates and identified a total of 5491 proteins for 24 h Ly6C^hi^CX_3_CR1^lo^ macrophages and 72 h Ly6C^lo^CX_3_CR1^hi^ macrophages, of which 4849 were common between two samples (Fig. 3A). A complete list of all identified proteins can be found in Table S1. Moreover, there were 5488 (99.9%) proteins quantified with more than one unique peptide and the median value of unique peptide number was 6 (Fig. S1A). The median value of protein sequence coverage was 22.5% (Fig. S1B). Then, the differentially expressed proteins were analyzed by Perseus software using t-test methods. In total, 211 proteins were found significantly (fold change ≥2, p value ≤ 0.05) regulated between 24 h Ly6C^hi^CX_3_CR1^lo^ macrophages and 72 h Ly6C^lo^CX_3_CR1^hi^ macrophages (Fig. 3B). From these, 137 proteins were up-regulated in 72 h Ly6C^lo^CX_3_CR1^hi^ macrophages compared with 24 h Ly6C^hi^CX_3_CR1^lo^ macrophages (marked as red dots), whereas 74 proteins were down-regulated (marked as green dots). Figure S2A shows a heatmap of the differentially expressed genes, and a list of these genes is available in Table S2.

Next, Gene Ontology (GO) enrichment analysis was performed using DAVID^22^. Endocytosis- and apoptotic cell clearance-related proteins were identified as significant up-regulated protein function group in 72 h Ly6C^lo^CX_3_CR1^hi^ macrophages (Fig. 3C), whereas translation-, response to Gram-positive bacterium or lipopolysaccharide-, or inflammatory response-related proteins were identified as down-regulated protein function group in 72 h Ly6C^lo^CX_3_CR1^hi^ macrophages (Fig. S2B). Phagocytosis of cellular debris after tissue injury has been shown to induce an anti-inflammatory phenotype in macrophages, which is important for the resolution of inflammation^6,7,23^. Our proteomic analysis showed that Ly6C^lo^CX_3_CR1^hi^ macrophages displayed upregulation of several endocytosis- or wound healing-related genes (e.g., *Alox15*, *Clec4f*, *Siglec1*, *Mrc1*, etc.) (Figs 3B and S2C), indicating that Ly6C^lo^CX_3_CR1^hi^ macrophages express a gene-expression signature associated with pro-resolving or alternatively activated phenotypes. 12/15-lipoxygenase (Alox15), which is restricted expressed on alternatively activated macrophages and macrophages populations participating in the resolution of inflammation^24,25^, has been shown to play anti-inflammatory and tissue repair roles^26,27^. CLEC4F is exclusively expressed on liver resident Kupffer cells and macrophages infiltrating into the liver^28^. It has been reported to be involved in the phagocytosis of desialylated platelets^29^. Sialoadhesin (Siglec1, Cd169) was initially defined as a macrophage adhesion receptor and was then shown to play important roles in receptor-mediated internalization process^30,31^. Macrophage mannose receptor 1 (Mrc1, Cd206) is a highly effective endocytic receptor and is considered as a marker for alternatively activated or M2 macrophages. Since these endocytosis- or wound healing-related genes are important indicators of pro-restorative macrophages, we focused on the expression of these genes for further validation. Consistent with proteomic results, quantitative PCR (qPCR) analysis confirmed significant increase in the expression of endocytosis- or wound healing-related genes *Alox15*, *Clec4f*, *Siglec1*, and *Mrc*1 in 72 h Ly6C^lo^CX_3_CR1^hi^ macrophages compared with 24 h Ly6C^hi^CX_3_CR1^lo^ macrophages (Fig. 3D).

Additionally, we selected a down-regulated protein associated with immune response for validation. Cathepsin G (Ctsg) has been shown to play important roles in antifungal immunity and endotoxic shock^32^. We performed western blot of the down-regulated protein, and found that marked decreased expression of Cathepsin G in the 72 h Ly6C^lo^CX_3_CR1^hi^ macrophages compared with the 24 h Ly6C^hi^CX_3_CR1^lo^ macrophages (Fig. S3A,B), which was consistent with proteomic data.

### Identification of Alox15 as a specific marker for Ly6C^lo^CX_3_CR1^hi^ macrophages

**Figure 4:**
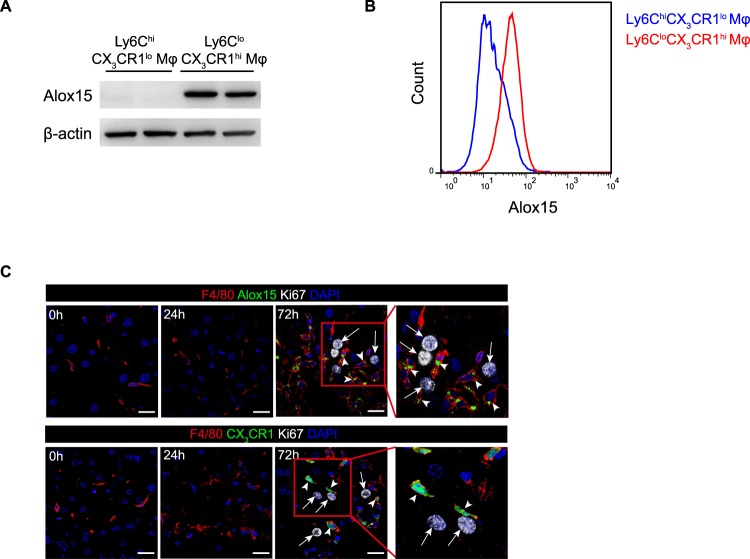
Identification of Alox15 as a specific marker for Ly6C^lo^CX_3_CR1^hi^ macrophages. (**A**) Western blot analysis for Alox15 expression in 24 h Ly6C^hi^CX_3_CR1^lo^ and 72 h Ly6C^lo^CX_3_CR1^hi^ macrophages. Full-length blots are shown in Fig. S3C. (**B**) Flow cytometric analysis for Alox15 expression in 24 h Ly6C^hi^CX_3_CR1^lo^ and 72 h Ly6C^lo^CX_3_CR1^hi^ macrophages. Cell populations from five mice were pooled to form one group. (**C**) Alox15^+^CX_3_CR1^hi^ macrophages were in close proximity to proliferating hepatocytes. White arrows indicate proliferating hepatocytes labeled with Ki67, and arrowheads indicate macrophages labeled with F4/80 and Alox15 in WT mice (upper panel) or F4/80 in *Cx3cr1*^*GFP*/+^ mice (lower panel). Scale bars, 20μm. Data shown are representative of three independent experiments.

Notably, Alox15 was significantly up-regulated protein in the Ly6C^lo^CX_3_CR1^hi^ macrophages relative to the Ly6C^hi^CX_3_CR1^lo^ macrophages, after using two-tailed t-test and Benjamini-Hochberg adjustment (Fig. 3B, Table S4). Indeed, the expression of Alox15 was restricted to Ly6C^lo^CX_3_CR1^hi^ macrophages but not Ly6C^hi^CX_3_CR1^lo^ macrophages, revealed by LC-MS/MS. Further confirmation of the unique expression of Alox15 was achieved by qPCR, Western blotting, flow cytometry and immunofluorescence staining (Figs 4A–C, S3C). Thus, we identified Alox15 as a specific marker for Ly6C^lo^CX_3_CR1^hi^ macrophages. Importantly, using *in situ* fluorescence labeling of Alox15 and CX_3_CR1-fluorescent reporter mice, we observed that the localization of Alox15^+^ and CX_3_CR1^hi^ macrophages was often adjacent to proliferating hepatocytes (Fig. 4C), suggesting that Ly6C^lo^CX_3_CR1^hi^ macrophages may exert positive effects on hepatocyte proliferation.

### Ly6C^lo^CX_3_CR1^hi^ macrophages directly accelerate hepatocyte proliferation *in vitro*

**Figure 5:**
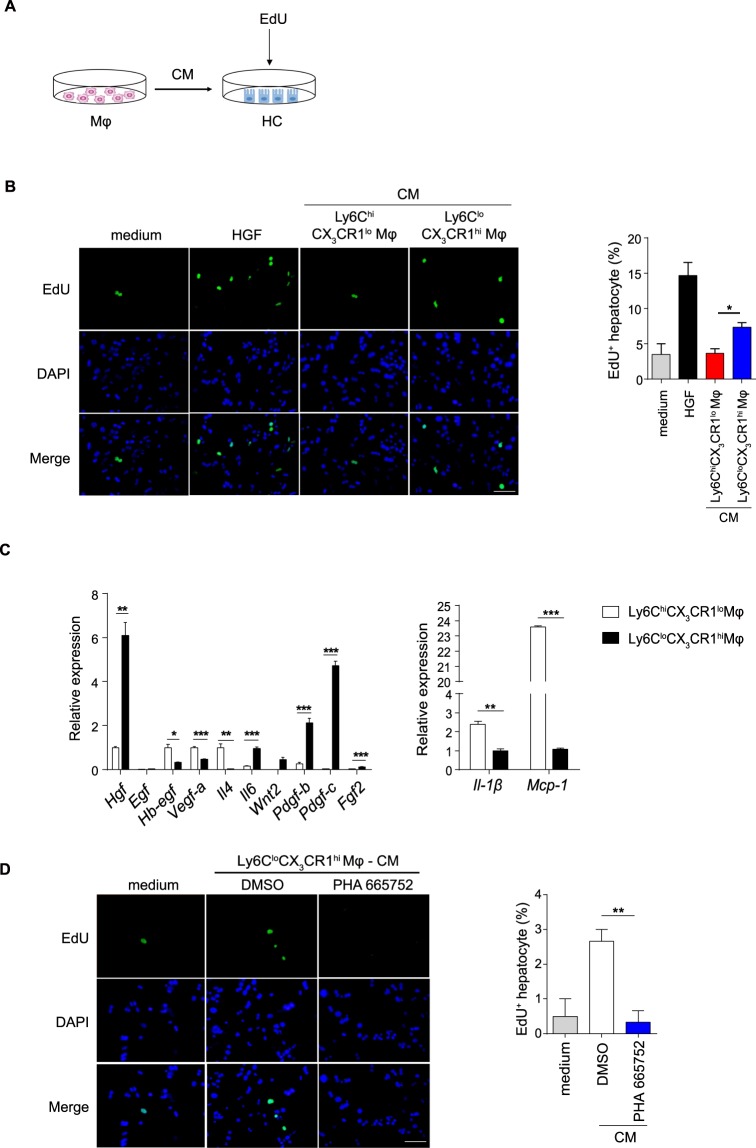
Ly6C^lo^CX_3_CR1^hi^ macrophages directly accelerate hepatocyte proliferation *in vitro*. (**A**) Schematic of the experimental design. Normal hepatocytes (HCs) were co-cultured with the CM from 24 h Ly6C^hi^CX_3_CR1^lo^ macrophages or 72 h Ly6C^lo^CX_3_CR1^hi^ macrophages. (**B**) Representative images of hepatocytes pulsed with EdU and the quantification of hepatocyte proliferation are shown. Scale bar, 50 μm. (**C**) Differential expression of the indicated genes measured by qPCR in 24 h Ly6C^hi^CX_3_CR1^lo^ macrophages and 72 h Ly6C^lo^CX_3_CR1^hi^ macrophages, presented relative to *Gapdh*. (**D**) Hepatocytes were co-cultured with the CM from 72 h Ly6C^lo^CX_3_CR1^hi^ macrophages or supplemented with the c-Met kinase inhibitor PHA665752 (2.5 μM). Representative images of hepatocytes pulsed with EdU (left panel) and the quantification of hepatocyte proliferation (right panel) are shown. Scale bar, 50 μm. The data shown are representative of at least two independent experiments (n = 3/group). The results represent means ± SEM (*p < 0.05, **p < 0.01, ***p < 0.001).

To validate the role of Ly6C^lo^CX_3_CR1^hi^ macrophages in hepatocyte proliferation, we performed a macrophage-hepatocyte co-culture system *in vitro* (Fig. 5A). Conditioned medium (CM) from 24 h Ly6C^hi^CX_3_CR1^lo^ macrophages failed to support hepatocyte proliferation, whereas CM from 72 h Ly6C^lo^CX_3_CR1^hi^ macrophages led to a significant increase in the number of EdU^+^ hepatocytes (Fig. 5B). These results suggest that Ly6C^lo^CX_3_CR1^hi^ macrophage may mediate hepatocyte proliferation through secretory signals.

Having uncovered that Ly6C^lo^CX_3_CR1^hi^ macrophages specifically stimulated hepatocyte proliferation, we wanted to identify which Ly6C^lo^CX_3_CR1^hi^ macrophage-derived hepatotropic cytokines were involved in liver regeneration. We performed a candidate-based screen of established hepatocyte growth factors by qPCR^33–36^. qPCR analysis identified marked increased expression of *Hgf*, *Il-6*, *Wnt2*, *Pdgf-b*, *Pdgf-c*, *Fgf2* accompanied by decreased expression of the pro-inflammatory genes *Il1β* and *Mcp-1* in the Ly6C^lo^CX_3_CR1^hi^ macrophages compared with the Ly6C^hi^CX_3_CR1^lo^ macrophages (Fig. 5C). Among these genes, the mRNA levels of HGF were the most significantly up-regulated (Fig. 5C). Thus, we speculated that Ly6C^lo^CX_3_CR1^hi^ macrophage-derived HGF might be involved in the pro-regeneration effect. To examine this hypothesis, we stimulated the cultured hepatocytes with conditioned medium from Ly6C^lo^CX_3_CR1^hi^ macrophages supplemented with the c-Met kinase inhibitor given that c-Met is the only known high-affinity receptor for HGF^37,38^. The addition of the c-Met kinase inhibitor in conditioned medium from Ly6C^lo^CX_3_CR1^hi^ macrophages decreased EdU incorporation (Fig. 5D), indicating that the proliferative actions of Ly6C^lo^CX_3_CR1^hi^ macrophages are mediated, in part, via the HGF/c-Met pathway.

### Selective depletion of Ly6C^lo^CX_3_CR1^hi^ macrophages delays liver regeneration and repair *in vivo*

**Figure 6:**
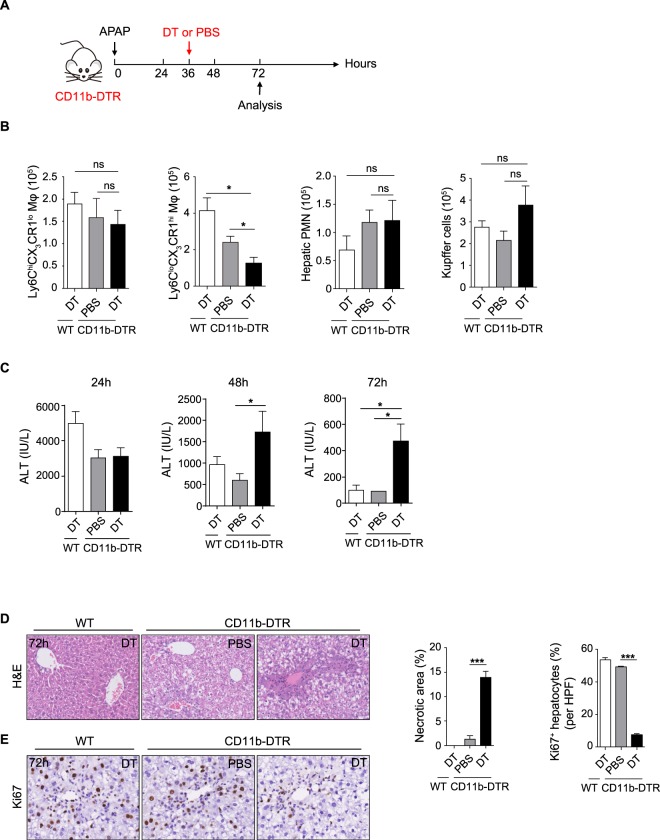
Selective depletion of Ly6C^lo^CX_3_CR1^hi^ macrophages delays liver regeneration and repair *in vivo*. (**A**) Schematic of the experimental design. WT and CD11b-DTR mice were challenged with APAP and were given DT or PBS at 36 h after APAP injection. (**B**) Flow cytometric analysis of distinct hepatic monocyte-derived macrophage populations, Kupffer cells and neutrophils at 72 h. (**C–E**) Serum ALT levels at the indicated time points (**C**), histological characterization (**D**), and IHC staining for Ki67 per high-powered field (HPF) (**E**) in liver sections at 72 h were evaluated. The data shown are representative of two independent experiments (n = 3–5/group). The results represent means ± SEM (*p < 0.05, **p < 0.01, ***p < 0.001).

To further confirm the role of Ly6C^lo^CX_3_CR1^hi^ macrophages in liver regeneration and repair *in vivo*, a well-described selective macrophage depletion strategy in CD11b- DTR transgenic mice was used^10^. We punctually ablated Ly6C^lo^CX_3_CR1^hi^ macrophages in CD11b-DTR mice during the resolution phase of liver injury, which is when Ly6C^lo^CX_3_CR1^hi^ macrophages predominate (Fig. 6A). Using this strategy, we observed that the number of hepatic Ly6C^lo^CX_3_CR1^hi^ macrophages was specifically reduced (Fig. 6B). Consistent with previously reports^10^, there was no significant reduction in the number of neutrophils and Kupffer cells after DT treatment, which may be due to the insensitivity of these cells to DT. Selective ablation of Ly6C^lo^CX_3_CR1^hi^ macrophages resulted in higher serum levels of ALT, more necrotic areas and a significantly lower number of Ki67^+^ proliferating hepatocytes during the resolution phase compared to the control mice (Fig. 6C–E). Collectively, these results strongly suggest that Ly6C^lo^CX_3_CR1^hi^ macrophages are required for optimal liver regeneration and repair.

## Discussion

Our exploratory proteomic studies of distinct macrophage populations from different phases provide evidence for better understanding the functional specialization of distinct macrophage subsets during the resolution of inflammation. Based on the proteomic results, we speculated that, unlike 24 h Ly6C^hi^CX_3_CR1^lo^ macrophages, 72 h Ly6C^lo^CX_3_CR1^hi^ macrophages may mediate wound healing and tissue repair. Indeed, recent studies have showed that Ly6C^hi^CX_3_CR1^lo^ macrophages aggravated APAP hepatotoxicity and inhibition of monocyte recruitment by blocking CCL2 or CCR2/CCR5 attenuated liver damage^39^. Here, we demonstrated the specific role of Ly6C^lo^CX_3_CR1^hi^ macrophages in the resolution of APAP-induced liver injury. The macrophage-hepatocyte co-culture experiments suggested that Ly6C^lo^CX_3_CR1^hi^ macrophages could specifically induce hepatocyte proliferation. Furthermore, specific ablation of Ly6C^lo^CX_3_CR1^hi^ macrophages delayed liver repair. Thus, our results provide evidences of a certain macrophage subpopulation at resolution phase of inflammation that directly induce tissue repair and regeneration, with important implications for treating inflammatory conditions.

In our study, we compared two different macrophage populations at different time points to analyze their gene expression profile. Since the two macrophage subsets predominated at different stages of inflammation and it was difficult to isolate enough cells at the same time points, we did not provide a more direct comparison about the molecular phenotype of these two macrophage subsets. However, based on previous reports^21,39^, the comparison between macrophages at different time points may also represent a reasonable approximation. In addition, it has been reported that Ly6C^hi^CX_3_CR1^lo^ macrophages can convert to Ly6C^lo^CX_3_CR1^hi^ macrophages during the resolution phase^20,21^. Thus, the comparison of 24 h Ly6C^hi^CX_3_CR1^lo^ macrophages and 72 h Ly6C^lo^CX_3_CR1^hi^ macrophages may also show the functional changes of macrophages before and after phenotypic transition.

Our comparative proteomic analysis indicated that 72 h Ly6C^lo^CX_3_CR1^hi^ macrophages exhibited a reparative protein expression profile. Furthermore, Alox15 was the most strongly up-regulated protein in Ly6C^lo^CX_3_CR1^hi^ macrophages and has been identified as a specific marker for Ly6C^lo^CX_3_CR1^hi^ macrophages by LC-MS/MS and subsequent validations. Previous studies have shown that Alox15 was restricted expressed in certain macrophage populations including alternatively activated macrophages and macrophages participating in the resolution of inflammation^24,40,41^. Consistent with these findings, our proteomic data and subsequent validation suggest that Alox15 was a specific marker for Ly6C^lo^CX_3_CR1^hi^ macrophages, which exhibited a pro-resolving profile. Furthermore, several lines of evidence suggest that Alox15 plays an anti-inflammatory and tissue-repair role in various contexts. Deletion of Alox15 leads to exacerbation of inflammation and tissue damage during chronic inflammatory disorders such as arthritis^42^. Alox15 can also contribute to generation of pro-resolving lipid mediators such as lipoxin A4, resolving E1, and protectin D1, which are responsible for resolution of inflammation^43–45^. Recent studies have identified Alox15 as a central factor orchestrating the sorting of apoptotic cells (ACs) and the clearance of ACs was confined to a population of Alox15-expressing, alternatively activated resident macrophages^27^. Thus, it is tempting to speculate that Alox15 is a major factor mediating tissue repair in macrophages. Further studies will be required to determine the specific contribution of Alox15 in Ly6C^lo^CX_3_CR1^hi^ macrophages to wound healing process.

Additionally, *in vitro* co-culture system suggested that 72 h Ly6C^lo^CX_3_CR1^hi^ macrophages directly stimulated hepatocyte proliferation. Selective depletion of this population in CD11b-diphtheria toxin receptor mice further confirmed the key role of Ly6C^lo^CX_3_CR1^hi^ macrophages in liver repair and regeneration. Although we observed that the pro-regenerative effects of Ly6C^lo^CX_3_CR1^hi^ macrophages on hepatocytes relied on the HGF/c-Met pathway, we cannot rule out the possibility that additional factors other than HGF might contribute to the pro-resolving effects on macrophages.

## Methods

### Mice

All animal procedures were approved by the Institutional Animal Care and Utilization Committee at Beijing Institute of Lifeomics. C57BL/6 wild-type (WT) mice were purchased from Charles River in Beijing (Vital River). CD11b-DTR mice were kindly provided by Dr. Honglin Wang (Shanghai Jiao Tong University). *Cx3cr1*^*gfp*/+^ mice were generously provided by Dr. Zhihua Liu (Institute of Biophysics, Chinese Academy of Sciences). All mice were maintained in our specific pathogen-free facilities. Sex- and age- matched controls were used, and all experiments were performed in accordance with the Institutional Animal Care and Utilization Committee-approved protocols.

For selective macrophage depletion, CD11b-DTR mice were injected intravenously (i.v.) with 30 ng/g body weight of DT (List Biological Labs) at 36 h post APAP challenge.

### APAP-induced hepatotoxicity and assays for liver injury

Mice were fasted for 16 hours and injected intraperitoneally (i.p.) with acetaminophen (Sigma Aldrich) at 400 mg/kg. Serum alanine aminotransferase (ALT) levels were evaluated by diagnostic kits. Liver specimens from APAP-challenged mice were fixed in 4% paraformaldehyde and embedded with paraffin for hematoxylin and eosin (H&E) staining and immunohistochemistry analyses. The sections were stained with H&E or with monoclonal Rabbit anti-mouse Ki67 (clone SP6, ab16667, Abcam). Necrotic areas and percentage of Ki67-positive cells were quantified by ImageJ software.

### Primary hepatic cell isolation

Liver leukocytes were isolated as previously described^15^. After *in situ* two-step collagenase perfusion, the livers were homogenized and filtered through a 70 μm nylon mesh. The cell suspension was centrifuged for 5 minutes at 500 g, the cell pellet was then re-suspended in 15 ml 35% Percoll (GE Healthcare) containing 100 U/ml heparin and was centrifuged for 15 minutes at 500 g. The resulting cell pellet containing leukocytes was then lysed for erythrocytes by 3-min incubation with red blood cell lysis solution (BD Biosciences). For hepatic macrophage purification, isolated hepatic leukocytes were stained for cell-surface markers. Then, distinct hepatic macrophage subpopulations (F4/80^lo^CD11b^hi^Ly6G^−^Ly6C^hi^CX_3_CR1^lo^ or F4/80^lo^CD11b^hi^Ly6G^−^Ly6C^lo^CX_3_CR1^hi^) were sorted by FACSAria III (BD Biosciences).

For primary mouse hepatocytes isolation, liver perfusion and digestion were performed as described above. Following digestion, the liver was dissociated and filtered to obtain single cell suspensions. Hepatocytes were collected by centrifugation for 3 minutes at 50 g.

### Flow cytometry and FACS sorting

Hepatic leukocytes were incubated with Fc blocking reagent (CD16/32, eBioscience) for 20 minutes followed by incubation with fluorescently-conjugated antibodies directed against mouse (all from eBioscience unless specified otherwise): CD45 (30-F11), F4/80 (BM8), CD11b (M1/70), Ly6C (HK1.4), CD115 (AFS98), CCR2 (475301, R&D systems), CX_3_CR1 (SA011F11, Biolegend), Ly6G (1A8, BD Biosciences), Gr-1(RB6-8C5) and CD11c (N418). CD68 (FA-11, AbD Serotec) was stained extracellularly and subsequently intracellularly using Fixation-and-Permeabilization buffers (eBioscience) according to the manufacturer’s protocol. Experiments were performed using the LSRFortessa cell analyzer (BD Biosciences) and the acquired data were analyzed with FlowJo software (Tree Star). For cell sorting, FACSAria III (BD Biosciences) were used and the purity of sorted cells was routinely more than 95%.

### Sample preparation for LC-MS/MS analysis

FACS-sorted cells were lysed by 8 M urea and the cell debris was pelleted by centrifugation at 10000 g for 5 min. Then, the clarified lysate was transferred into a new vial. The lysate was diluted 1:8 with 50 mM NH_4_HCO_3_ and reduced by DTT (10 mM). Then it was alkylated with 50 mM IAA. The digestion was performed with trypsin (1:50) at 37 °C overnight. The resulting peptides were acidified with 0.5% formic acid, and the supernatant was collected. In each experiment, approximately 15 μg of peptides measured by nanodrop A280 method were loaded for fractionation. The amount of peptides was controlled to be equal between each replicate. The first dimension separation by reversed phase chromatography was performed using in-house C18 (3μm, 150 Å, Agela) 200 μl tip. The peptide mixtures were sequentially separated with gradient elution buffer (6, 9, 12, 15, 18, 21, 24, 30 and 35% buffer B) and then cross combined into six fractions and dried. The peptides were analyzed by Q-Exactive mass spectrometer and Orbitrap Fusion coupled to an Easy-nLC 1000 system (Thermo Fisher Scientific). Samples were dissolved in 0.1% formic acid and loaded onto a 12 cm reversed phase column (150 nm id) packed with C18 resin (1.9 μm). A binary solvent system (buffer A: 0.1% formic acid in water; buffer B: 0.1% formic acid in acetonitrile) was used for peptide separation. The gradient was set as follows: 5–8% B for 8 min, 8–22% B for 50 min, 22–32% B for 12 min, 32–90% B for 1 min, and 90% B for 7 min. The constant flow rate was 300 nl/min. Data dependent acquisition mode was used to automatically pick peptides for MS2 fragmentation. When Q-Exactive was used, top 20 method was used and the dynamic exclusion duration was set 18 s. In MS1, the scan range was set 300–1400 m/z, and the resolution of MS1 was set 70,000. The AGC target was 3e6, and the maximum injection time was 60 ms. In MS2, the resolution of MS2 was set 15,000. The AGC target was 5e4, and the maximum injection time was 80 ms. When Orbitrap Fusion was used, the peptides were cross combined into three fractions. Top speed method with a cycle time of 3 s was used and the dynamic exclusion duration was set 18 s. In MS1, the scan range was set 300–1400 m/z, and the resolution of MS1 was set 120,000. The AGC target was 5e5, and the maximum injection time was 100 ms. In MS2, iontrap was used. The AGC target was 5e3, and the maximum injection time was 35 ms.

### Data analysis of proteomic raw files

Raw MS data were processed using Maxquant (version 1.5.2.8) software^46^. In this software, the identified peptides were divided into two cases, unique peptides which are unique to the specific proteins and razor peptides which are found in more than one protein. As previously reported^47^, MaxQuant resolves this issue by collapsing all proteins that cannot be distinguished based on the identified peptides into protein groups. We used the default setting of the software that razor peptides contribute only to the quantification of the protein with the larger number of identifications. ‘Label free quantification’ was processed with Maxquant software. It was based on MS1 quant, and all the peak isotopes were used for MS1 quant. Normalization was performed using a solution named MaxLFQ based on peptide ion intensity. After summing up intensities with normalization factors as free variables, the software determined their quantities via a global optimization procedure based on achieving the least overall proteome variation. It was done purely from the data obtained and without the addition of external quantification standards or reliance on a fixed set of “housekeeping” proteins. A final protein level quantification value was generated by summing all identified peptide intensities. Detailed algorithm and protocols referred to previous reports^47,48^. The Mus musculus protein database (2018-05-29) was downloaded from Uniprot and only 16,978 canonical sequences which were annotated as “reviewed” were used for database searching. Trypsin was selected as the proteolytic enzyme. Two missed cleavages was allowed. Cysteine carbamidomethylation was set as the fixed modification. N-terminal acetylation, methionine oxidation were set as the variable modifications. The false discovery rate was set ≤1% at spectra level and protein level. Label-free quantitation was performed. The database search results were then processed with Perseus (1.5.8.5) software^49^. After removing the reversed and contaminating proteins (such as BSA), the LFQ values were log_2_ transformed and used for comparison between different experiment conditions. T-test was performed to obtain the differential expressed proteins with Perseus (1.5.8.5) software.

### Western blot analysis

Macrophage protein extracts were prepared according to standard protocols. Cell lysates were separated by 8% or 10% SDS-PAGE and transferred to polyvinylidene difluoride membranes (Millipore). The following antibodies were used: Cathepsin G (ab197354, Abcam), Alox15 (sc-32940, Santa Cruz) and β-actin (A5441, Sigma Aldrich).

### Quantitative RT-PCR

Total RNA was extracted using RNeay Mini Kit (Qiagen) according to the manufacturer’s protocol and cDNA was made using Reverse Transcription kit (Promega). The cDNA was used for quantitative qPCR analysis on an iCycler iQ5 Real-Time PCR detection system (BioRad). The expression of target gene was normalized to the expression of the housekeeping gene, *Gapdh*. Relative gene expression was calculated using the standard 2^−ΔΔCt^ method. A full list of the primer sequences is available in Table S3.

### Immunofluorescence

Mouse liver tissues were fixed in 4% paraformaldehyde and then embedded in OCT (Sakura). 5 μm Frozen sections were prepared using a Cryotome FSE cryostat (Thermo-Fisher Scientific). The tissue sections were incubated in the blocking buffer (5% donkey serum, 0.3% Triton-X 100 in PBS) at room temperature for 1 hr followed by the staining with primary antibodies. The following primary antibodies were used: Rat anti-mouse F4/80 (clone CI:A3-1, ab6640, Abcam), Rabbit anti-mouse Ki67 (clone SP6, ab16667, Abcam), goat anti-mouse Alox15 antibody (clone H-235, sc-32940, Santa Cruz). Then slides were washed and incubated for 1 h with the following secondary antibodies: donkey anti-rat Alexa Fluor 488, donkey anti-goat Alexa Fluor 594 and donkey anti-rabbit Alexa Fluor 647 (Jackson ImmunoResearch Laboratories). Sections were counterstained with 4′6-diamidino-2-phenylindole dihydrochloride (DAPI) before being mounted. All immunofluorescence staining was performed in the dark. Imaging was performed using a Zeiss LSM 880 and images were processed using Zeiss ZEN software.

### Determination of hepatocyte proliferation by EdU labeling

Primary mouse hepatocytes were isolated from WT mice. Ly6C^hi^CX_3_CR1^lo^ macrophages and Ly6C^lo^CX_3_CR1^hi^ macrophages were isolated at the indicated time points. Conditioned medium (CM) was collected from 2 × 10^5^ Ly6C^hi^CX_3_CR1^lo^ or Ly6C^lo^CX_3_CR1^hi^ macrophages, filtered through a 0.22 μm filter, and added to 1 × 10^4^ hepatocytes. Hepatocytes were treated with macrophage-derived CM or HGF (50 ng/ml, Peprotech) for 12 h, followed by EdU (5-ethynyl-2′-deoxyuridine, 20 μM) pulsing for an additional 36 h. In another series of experiments, isolated hepatocytes were treated with Ly6C^lo^CX_3_CR1^hi^ macrophage-derived CM supplemented with or without the c-Met kinase inhibitor PHA665752 (2.5 μM, R&D systems). Hepatocytes undergoing DNA synthesis were visualized using the EdU Imaging Kit (Life Technologies). Imaging was performed using Olympus IX71 inverted fluorescence microscopes and EdU-positive cells were quantified by ImageJ software.

### Statistical analysis

Statistical analysis was performed with GraphPad Prism 5 software. Data are presented as mean ± SEM. Statistical significance was determined by unpaired, two-tailed, Student’s t test; p < 0.05 was considered statistically significant. Group allocation and outcome assessment was performed in a blinded manner. No exclusion criteria were applied, and all samples were included in data analysis.

## Supplementary information

Supplementary Information

Supplementary Information-Tables

## Data Availability

The mass spectrometry proteomics data have been deposited to the ProteomeXchange Consortium via the PRIDE partner repository with the dataset identifier PXD011958.
