# Supplementary material for: Proteomic analysis reveals a protective role of specific macrophage subsets in liver repair
Source: Sci Rep. 2019 Feb 27;9:2953. doi: 10.1038/s41598-019-39007-6 (PMC6393665; doi:10.1038/s41598-019-39007-6)
Supplement: Supplementary file 1 — Supplementary Information [file 41598_2019_39007_MOESM1_ESM.pdf]

Proteomic analysis reveals a protective role of specific macrophage subsets in liver repair

Wenting Yang[1], Xinyuan Zhao[1], Yuandong Tao[1], Yan Wu[1], Fuchu He[1,*], and Li Tang[1,2,*]

[1]State Key Laboratory of Proteomics, National Center for Protein Sciences, Beijing. Beijing Proteome Research Center, Beijing Institute of Lifeomics, Beijing 102206, P.R. China

[2]Department of Biochemistry and Molecular Biology, Anhui Medical University, Hefei, Anhui Province, 230032, P.R. China

Correspondence:

*(F.H.) E-mail: hefc@nic.bmi.ac.cn; Tel and Fax: +86-10-68171208

*(L.T.) E-mail: tangli08@aliyun.com; Tel and Fax: +86-10-61777091

Figure S1

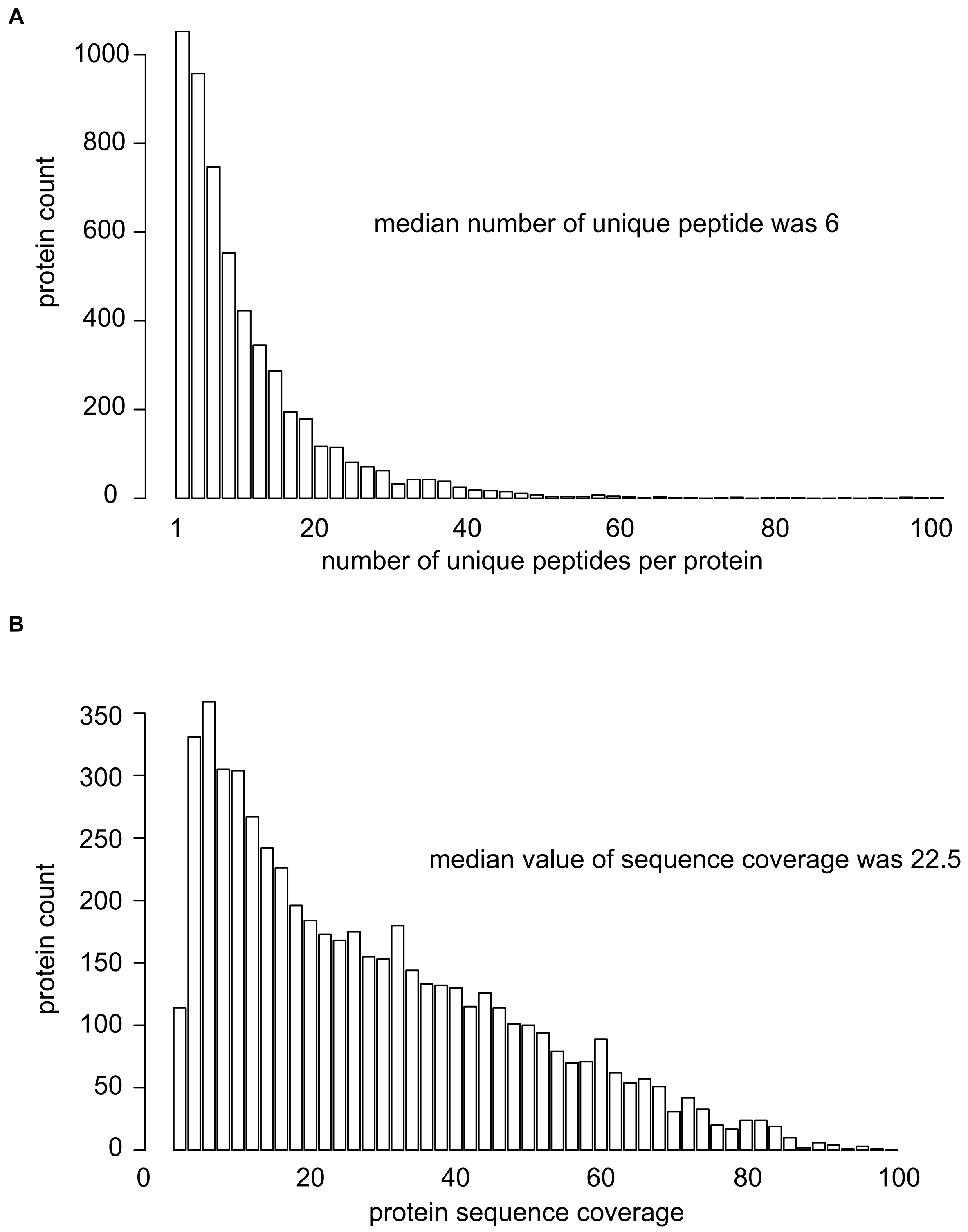

Figure S1. Histogram of the unique peptides and sequence coverage for the quantified proteins.

(A) Histogram of the unique peptides for the quantified proteins. The median number of unique peptide was 6.

(B) Histogram of the sequence coverage (%) for the quantified proteins. The median value of protein sequence coverage was 22.5.

Figure S2

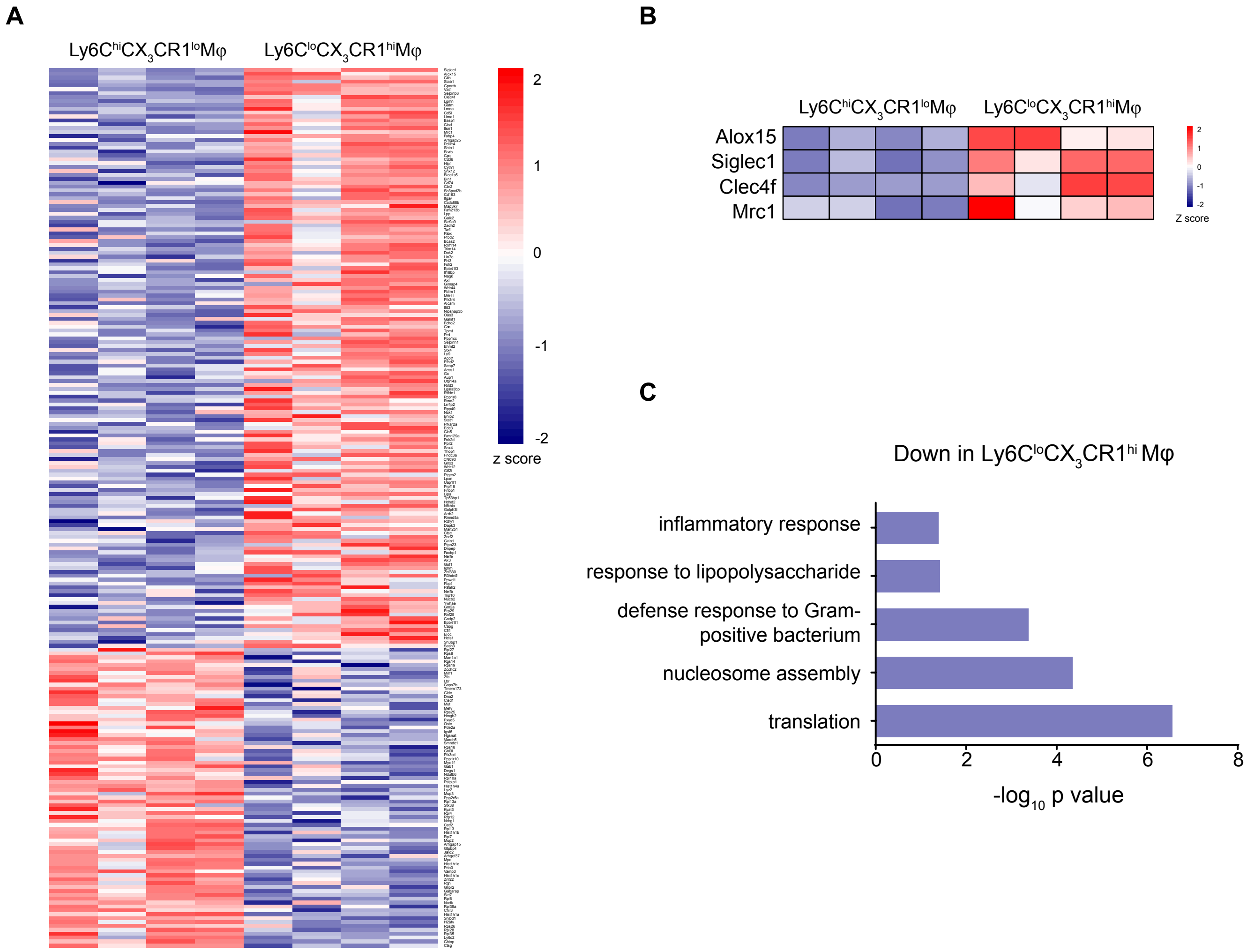

Figure S2. Proteomic analysis of distinct macrophage subsets from distinct phases in APAP-induced liver injury.

(A) Heatmap showing the differentially expressed genes in 24 h Ly6C^{hi}CX$_3$CR1^{lo} macrophages as compared to 72 h Ly6C^{lo}CX$_3$CR1^{hi} macrophages. Row-based Z score normalized.

(B) Heatmap showing differential expression of the indicated genes in 24 h Ly6C^{hi}CX$_3$CR1^{lo} macrophages as compared to 72 h Ly6C^{lo}CX$_3$CR1^{hi} macrophages.

(C) Gene Ontology (GO) enrichment analysis of down-regulated genes in 72 h Ly6C^{lo}CX$_3$CR1^{hi} macrophages was classified by their biological functions.

Figure S3

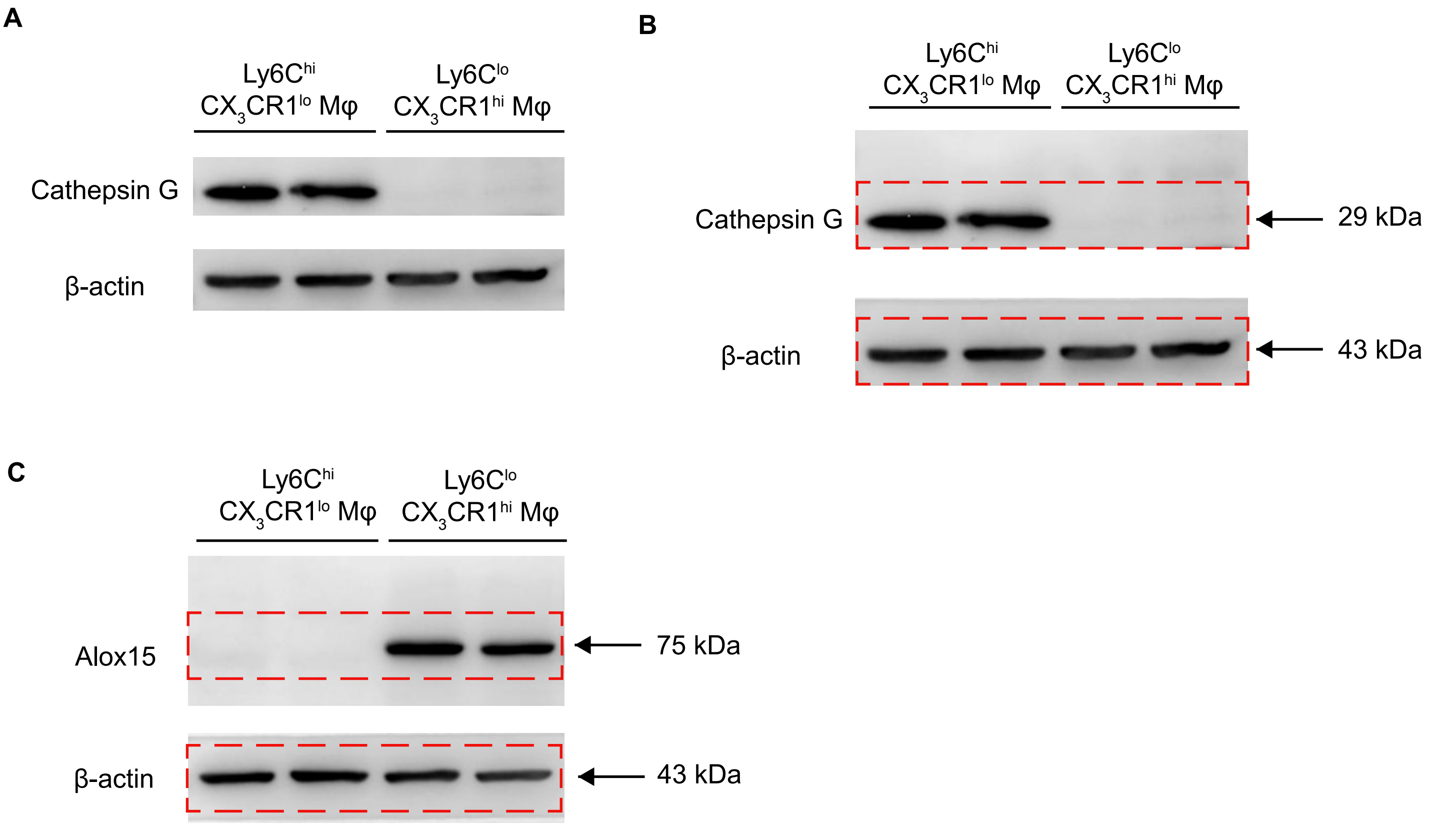

Figure S3. Western blot analysis for the differentially expressed proteins.
(A) Western blot analysis for the indicated down-regulated proteins.
(B) Full-length western blots of **Figure S3A**. The Red dotted lines show the cropping locations.
(C) Full-length western blots of **Figure 4A**.
